# The role of PD-L1 expression as a predictive biomarker: an analysis of all US Food and Drug Administration (FDA) approvals of immune checkpoint inhibitors

**DOI:** 10.1186/s40425-019-0768-9

**Published:** 2019-10-26

**Authors:** Andrew A. Davis, Vaibhav G. Patel

**Affiliations:** 1 Division of Hematology and Oncology, Department of Medicine, Northwestern University Feinberg School of Medicine, Chicago, IL USA; 2 Robert H. Lurie Comprehensive Cancer Center of Northwestern University, Chicago, IL USA; 3 Division of Hematology and Medical Oncology, Tisch Cancer Institute, Icahn School of Medicine at Mount Sinai, 1 Gustave Levy Library Place, New York, NY 10029 USA

**Keywords:** Immunotherapy, Immune checkpoint inhibitors, PD-L1, FDA

## Abstract

The development of immune checkpoint inhibitors has changed the treatment paradigm for advanced cancers across many tumor types. Despite encouraging and sometimes durable responses in a subset of patients, most patients do not respond. Tumors have adopted the PD-1/PD-L1 axis for immune escape to facilitate tumor growth, which can be leveraged as a potential target for immune checkpoint inhibitors. On this basis, PD-L1 protein expression on tumor or immune cells emerged as the first potential predictive biomarker for sensitivity to immune checkpoint blockade. The goal of our study was to evaluate PD-L1 as a predictive biomarker based on all US Food and Drug Administration (FDA) drug approvals of immune checkpoint inhibitors. We evaluated the primary studies associated with 45 FDA drug approvals from 2011 until April 2019. In total, there were approvals across 15 tumor types. Across all approvals, PD-L1 was predictive in only 28.9% of cases, and was either not predictive (53.3%) or not tested (17.8%) in the remaining cases. There were 9 FDA approvals linked to a specific PD-L1 threshold and companion diagnostic: bladder cancer (*N* = 3), non-small cell lung cancer (N = 3), triple-negative breast cancer (*N* = 1), cervical cancer (N = 1), and gastric/gastroesophageal junction cancer (N = 1) with 8 of 9 (88.9%) with immune checkpoint inhibitor monotherapy. The PD-L1 thresholds were variable both within and across tumor types using several different assays, including approvals at the following PD-L1 thresholds: 1, 5, and 50%. PD-L1 expression was also measured in a variable fashion either on tumor cells, tumor-infiltrating immune cells, or both. In conclusion, our findings indicate that PD-L1 expression as a predictive biomarker has limitations and that the decision to pursue testing must be carefully implemented for clinical decision-making.

## Introduction

Immune checkpoint inhibitors have changed the treatment landscape for many tumor types, particularly in the metastatic setting. Since the first Food and Drug Administration (FDA) approval in 2011, the pace of discovery has increased dramatically. While meaningful, durable responses are achieved in some patients, the majority of patients do not respond. Thus, predictive biomarkers of sensitivity and resistance to immune checkpoint blockade are needed. To date, the search for predictive biomarkers has been challenging given the dynamic interplay between these antibodies and the immune microenvironment and the heterogeneity of immune milieu in different tumor types [1]. The most frequently studied biomarker, PD-L1 protein expression on either tumor or immune cells, emerged early based on the mechanism of interaction between PD-1 and PD-L1 [2]. Specifically, PD-1 (CD279), predominantly expressed on surface of activated T and B lymphocytes, plays a vital role in maintaining peripheral and central immune cell tolerance by binding to its ligands, PD-L1 (B7-H1) and PD-L2 (B7-DC), and inhibiting peripheral T-cell activation [1]. PD-L1 is expressed on a variety of normal and immune cell types and is much more commonly present than PD-L2 [3]. Tumor cells have also adopted this PD-1/PD-L1 mechanism to suppress immune surveillance and facilitate tumor growth [2]. Thus, the use of immune checkpoint blockade particularly in patients with tumor and tumor-infiltrating immune cell population expressing PD-L1 has been of critical interest.

Herein, we systematically evaluated pivotal trials leading to all FDA approvals of immune checkpoint inhibitors from 2011 to 2019 and report the status of PD-L1 expression as a predictive biomarker. In addition, we carefully examined the FDA indications that were specifically linked to PD-L1 testing. Finally, we discuss the challenges of PD-L1 as a biomarker and offer future directions for biomarker investigation in the immunotherapy space.

## Methods

We examined all immune checkpoint inhibitor FDA approvals from the first approval in 2011 until April 1st 2019. Institutional Review Board (IRB) approval was waived for this retrospective study given that no patient-protected health information was utilized. In total, 45 approvals were found over this time period using the following FDA website:

https://www.fda.gov/drugs/resources-information-approved-drugs/hematologyoncology-cancer-approvals-safety-notifications. We then evaluated the primary articles from the specific clinical trial or trials that were linked to the FDA approval (Additional file 1: Table S1). For each study, the potential for PD-L1 as a predictive biomarker was examined, either with respect to predicting response of the approved drug as compared to chemotherapy or to predict treatment response at a higher threshold of PD-L1 expression compared to a lower threshold in single-agent studies. For each trial, we recorded the PD-L1 cutoff(s) studied, whether PD-L1 expression pertained to tumor and/or immune cells, the PD-L1 assay utilized, and whether the FDA approval was linked to a cutoff and/or assay. In addition, we reported whether the FDA approval was related to another predictive biomarker for response to immune checkpoint inhibitors.

Summative data were analyzed using categorical variables. Response data across trials were not compared given the variability of trial designs including single-arm vs. various comparator groups, different PD-L1 thresholds, and multiple tumor types included in our analyses.

## Results

In total, there were 45 FDA approvals from 2011 to April 2019. The pace of approvals increased over time with one in 2011, two in 2014, seven in 2015, seven in 2016, twelve in 2017, thirteen in 2018, and three thus far in 2019. The majority of the approvals were as a result of Phase II (22/45, 49%) and Phase III (20/45, 44%) clinical trials. Approvals included fifteen tumor types (NSCLC (*N* = 11), melanoma (*N* = 8), bladder (*N* = 5), renal (*N* = 2), head and neck (*N* = 2), colon (*N* = 2), hepatocellular (*N* = 2), small cell lung cancer (*N* = 2), Merkel-cell carcinoma (*N* = 2), squamous cell carcinoma of the skin (*N* = 2), Hodgkin’s lymphoma (*N* = 2) and the following with one each (breast, cervical, gastric/gastroesophageal junction (GEJ), primary mediastinal B-cell lymphoma, and one that was tissue agnostic). The most commonly approved immune checkpoint inhibitors included pembrolizumab (*N* = 18), nivolumab (*N* = 11), followed by atezolizumab (*N* = 5), ipilimumab with nivolumab (*N* = 3), ipilimumab (*N* = 2), durvalumab (*N* = 2), cemiplimab (*N* = 2), and avelumab (*N* = 2).

**Fig. 1:**
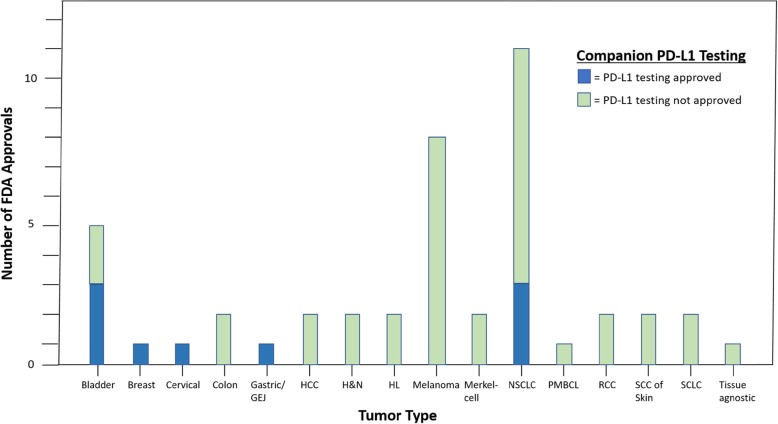
Number of immune checkpoint inhibitor FDA approvals by tumor type: The colors in the key denote whether PD-L1 testing was approved (blue) or not approved (green) as a companion diagnostic. Abbreviations: GEJ = gastro-esophageal junction; HCC = hepatocellular carcinoma; HL = Hodgkin’s Lymphoma; NSCLC = non-small cell lung cancer; PMBCL = primary mediastinal B-cell lymphoma; RCC = renal cell carcinoma; SCC = squamous cell carcinoma; SCLC = small cell lung cancer

With respect to PD-L1 status, nine FDA approvals were linked to PD-L1 testing (Table 1). Approvals related to PD-L1 status included the following tumor types: bladder cancer (*N* = 3), NSCLC (*N* = 3), triple-negative breast cancer (*N* = 1), cervical cancer (*N* = 1), and gastric/GEJ cancer (*N* = 1) (Fig. 1). The PD-L1 thresholds were variable both within and across tumor types and indications, including approvals at the following PD-L1 thresholds: 1, 5, and 50%. The type of cells expressing PD-L1 also varies by approval. For example, for NSCLC, approval was based on staining for PD-L1 on tumor cells. In contrast, the triple-negative breast cancer approval was based on tumor-infiltrating immune cells, and the cervical cancer approval used a composite proportion score of tumor and immune cells. Eight of nine approvals (89%) were for immune checkpoint inhibitor monotherapy. Furthermore, FDA indications were tied to different companion diagnostics, depending on the antibody used in the particular study leading to approval. These included SP142 (Ventana Medical Systems), SP263 (Ventana Medical Systems), and IHC 22C3 (Dako North America, Inc.).

**Table 1:** FDA approvals for immune checkpoint inhibitors linked to PD-L1 testing

Tumor Type	Drug	Mechanism	Approval Year	Comparator	Line of Therapy	PD-L1 Threshold	PD-L1 Tissue Testing	PD-L1 Cell Staining	Companion Diagnostic	Number of Patients	Endpoint for Approval	Results
NSCLC	Pembrolizumab	PD-1	2015	None	2nd	0.50	Fresh or archival for training Fresh for validation	TC	IHC 22C3	495 (182 training, 313 validation)	ORR	*Overall* 19.4% overall; *Training* 34.2% (TPS ≥50), 9.3% (TPS 1–49) 10.0% (TPS < 1) *Validation* 45.2% (TPS ≥50), 16.5% (TPS 1–49), 10.7% (TPS < 1)
NSCLC	Pembrolizumab	PD-1	2016	Docetaxel	2nd	0.01	Fresh or archival	TC	IHC 22C3	1034	OS	OS: *Pembrolizumab 2 mg/kg* HR 0.71 (95% CI, 0.58–0.88; *P* = 0.0008) *Pembrolizumab 10 mg/kg* HR 0.61 (0.49–0.75; *P* < 0.0001)
NSCLC	Pembrolizumab	PD-1	2016	Platinum-based chemotherapy	1st	0.50	Fresh or archival	TC	IHC 22C3	305 05	PFS, OS	PFS: HR 0.50 (95% CI, 0.37–0.68, *P* < 0.001) OS: HR 0.60 (95% CI, 0.41–0.89, *P* < 0.005)
Bladder	Atezolizumab	PD-L1	2016^a^	None	1st	0.05	Archival	IC	SP142	310	ORR	*Overall* 14.8% *PD-L1+* 26.0% *PD-L1-* 9.5%
Bladder	Pembrolizumab	PD-1	2017^a^	Chemotherapy of investigator’s choice	1st	0.10	Fresh or archival	TC + IC	IHC 22C3	542	ORR, OS	ORR: *Overall* 21.1% vs. 11.4% (chemotherapy) *CPS ≥ 10* 21.6% vs. 6.7% (chemotherapy) OS: *Overall* HR 0.73 (95% CI, 0.59–0.91, *P* = 0.002) *CPS ≥ 10* HR 0.57 (95% CI, 0.37–0.88, *P* = 0.005)
Bladder	Durvalumab	PD-L1	2017	None	2nd	0.25	Fresh or archival	TC + IC	SP263	191	ORR	*Overall* 17.8% *PD-L1+* 27.6% *PD-L1-* 5.1%
Gastric/GEJ	Pembrolizumab	PD-1	2017	None	3rd	0.01	Fresh or archival	TC + IC	IHC 22C3	259	ORR	*Overall* 11.6% *PD-L1+* 15.5% *PD-L1-* 6.4%
Cervical	Pembrolizumab	PD-1	2018	None	2nd	0.01	Fresh or archival	TC + IC	IHC 22C3	98	ORR	*Overall* 12.2% *PD-L1+* 14.6%^b^
Triple-negative breast cancer	Atezolizumab + nab-paclitaxel	PD-L1	2019	Nab-paclitaxel	1st	0.01	Fresh or archival	IC	SP142	451	ORR, PFS	ORR: *Overall* 56.0% vs. 45.9% (placebo arm) *PD-L1+* 58.9% vs. 42.6% (placebo arm) PFS: *Overall* HR 0.80 (95% CI, 0.69–0.92, *P* = 0.002) *PD-L1+* HR 0.62 (95% CI, 0.49–0.78, *P* < 0.001)

Abbreviations: *CPS* combined positive score, *NSCLC* non-small cell lung cancer, *GEJ* gastroesophageal junction, *IC* immune cells, *TC* tumor cells, *TPS* tumor proportion score

*CPS* number of PD-L1+ cells (tumor, lymphocytes, and macrophages) divided by total number of cells (tumor, lymphocytes, and macrophages), multiplied by 100

*TPS* number of PD-L1+ tumor cells divided by total number of tumor cells, multiplied by 100

^a^In 2018, companion PD-L1 testing approved as first-line for cisplatin-ineligible patients with locally advanced/metastatic urothelial carcinoma including Ventana SP142 (PD-L1 > 5%) treated with atezolizumab and Dako 22C3 assay CPS > 10 treated with pembrolizumab

^b^All 12 responses observed in patients with PD-L1+ tumors

**Fig. 2:**
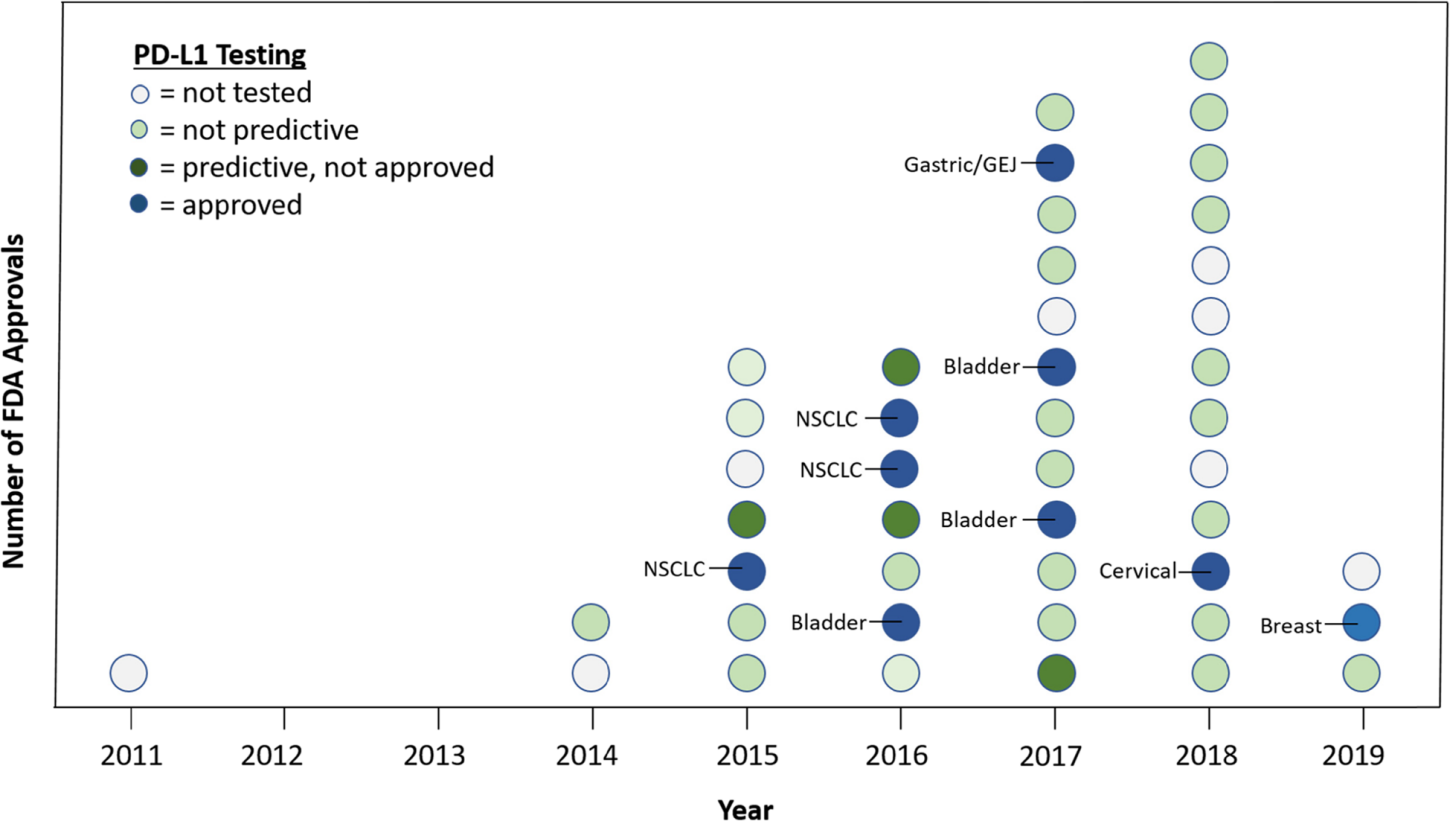
Number of immune checkpoint inhibitor FDA approvals by year: The colors in the key denote the predictiveness and approval status of PD-L1 status as a companion diagnostic. The labeled tumor types (in blue) represent approvals with PD-L1 testing as a companion diagnostic. Abbreviations: GEJ = gastroesophageal junction, NSCLC = non-small cell lung cancer

Across the 45 cases included, PD-L1 was predictive in 28.9% of the approvals and was either not predictive (53.3%) or not tested (17.8%) in the remaining cases (Fig. 2). The reporting of PD-L1 expression across studies was highly variable with the following types of cells examined: tumor cells (*N* = 22), tumor and immune cells (*N* = 10), immune cells (*N* = 2), tumor or immune cell (*N* = 1), not stated (*N* = 2), or not performed (*N* = 8). The only other predictive biomarker that was related to an approval was microsatellite-high (MSI-high)/mismatch repair-deficient status in three cases.

## Discussion

Based on the hypothesis that PD-L1 is a crucial protein for tumor immune escape and its presence indicates a potential target for immune checkpoint inhibitors, PD-L1 emerged as an early biomarker to be tested in immunotherapy clinical trials. In fact, more than 80% of pivotal trials that led to FDA approval had PD-L1 expression as a correlate. Despite the widespread investigation in the clinical trial setting, this study illustrates the imprecise nature of PD-L1 as a predictive biomarker. Specifically, PD-1 positivity predicted increased response in less than 30% of studies and importantly, only 20% of all approvals have companion PD-L1 diagnostic testing. Furthermore, the estimates of utility of PD-L1 biomarker may be exaggerated as our review only included “positive” trials that resulted in FDA approvals.

Several reasons may account for the heterogeneity in PD-L1 predictiveness. Firstly, as our findings highlight, there is a large variability amongst the included trials in terms of type of tissue tested (fresh vs. archival), type of PD-L1 assay, PD-L1 expression cutoffs, and type of cells (tumor vs. immune vs. both) tested for PD-L1 expression. This presents a significant challenge for pathologists and clinicians to decipher the various modes of testing and its application in routine clinical practice. Second, PD-L1 expression is regulated by several molecular pathways and by other immune cells in the tumor microenvironment and its ability to drive immunogenicity may be variable for different tumor types [4]. In animal model systems, early evidence suggests that PD-L1 expression on both tumor and immune cell may contribute to tumor evasion and inhibiting antitumor immunity across different tumor types [5]. The relative contribution of these cell components is likely context dependent. For example, one study in NSCLC patients treated with atezolizumab demonstrated objective response rates for high tumor cell PD-L1 and high immune cell PD-L1 of 40 and 22%, respectively, and that these populations were independent [6]. Third, PD-L1 expression has temporal and spatial heterogeneity [7] and can be altered with exposure to prior therapies [4].

Although PD-L1 testing has not delivered as a broadly applied biomarker, it holds value for certain tumor types as outlined in Table 1 and remains the most common immune-based biomarker in current clinical practice. In NSCLC, two large phase III studies revealed the superiority of pembrolizumab over chemotherapy in prolonging survival in platinum-refractory and chemotherapy-naïve patients harboring PD-L1 expression > 1% and > 50%, respectively [8, 9]. Despite its promise as a useful biomarker, in the first year after approval, PD-L1 testing in NSCLC was utilized in only approximately 11% of community practices [10]. Although the use of PD-L1 testing has increased over time since its approval [10], the true estimates in academic and community settings remain uncertain. In urothelial carcinoma, atezolizumab and pembrolizumab, were approved with their companion PD-L1 diagnostic testing, Ventana SP142 and Dako IHC 22C PharmDx Assay, respectively, for first-line platinum-ineligible patients. These approvals were based on superior clinical efficacy in PD-L1+ tumors, compared to PD-L1- tumors, in platinum-refractory patients. Specifically, atezolizumab improved ORR in PD-L1+ compared to PD-L1- tumors [11], while pembrolizumab demonstrated a survival benefit when compared to standard chemotherapy irrespective of PD-L1 status [12]. Durvalumab was also approved with its own PD-L1 diagnostic, Ventana SP263, for platinum-refractory patients, based on improved ORR; however, the use of this diagnostic was designated only as complementary. Despite the promising durable responses in many patients harboring PD-L1 expression, there was no correlation between degree of PD-L1 expression and response rate in these clinical trials [13]. Additionally, some patients without PD-L1 expression also demonstrated durable responses [13]. Thus, the clinical utility of PD-L1 in urothelial carcinoma at this time is rather limited. On the contrary, patients with heavily pre-treated gastric/GEJ and platinum-refractory cervical cancers who harbor PD-L1 expression can potentially benefit from immune checkpoint blockade as an additional form of therapy [14, 15]. Most recently, PD-L1 companion diagnostic testing was approved for first-line treatment of triple-negative breast cancer. This was based on phase III data, which showed improved PFS and ORR in patients receiving nab-paclitaxel with atezolizumab compared to nab-paclitaxel alone with clinical efficacy that was predominantly observed in patients with PD-L1+ tumors [16].

Our study has several limitations. First, we only included studies leading to FDA drug approval. Therefore, our analyses overestimated the predictive nature of PD-L1 as a biomarker. Second, given the variety of study designs, lines of therapy, and tumor types, we could not evaluate pooled outcome measures across studies. Lastly, we cannot define the basis of FDA for companion PD-L1 diagnostic testing approvals, as there were three studies that were predictive but not approved.

In addition to PD-L1 expression, an intensive search for novel predictive biomarkers for immune checkpoint blockade is taking place. One example is tumor mutational burden (TMB), which refers to the number of somatic mutations in tumors, tends to be higher in particular tumor types, such as melanoma, NSCLC, and urothelial carcinoma due to mutagenic exposures [17]. Recently, clinical trials for NSCLC and urothelial carcinoma indicate that TMB may in fact be predictive [18–20]. Additionally, TMB also appears to be independent of PD-L1 status [21]. However, some challenges for clinical implementation of TMB include defining uniform detection methods and appropriate thresholds for response by tumor type [22]. Other potential predictive biomarkers include T cell-inflamed gene expression profile (GEP) and tumor-infiltrating lymphocytes (TILs) [23, 24].

Collectively, our findings highlight the complexity of establishing uniform biomarkers for response to immune checkpoint inhibitors. Compared to matching a particular drug with a known genomic mutation, fusion, or protein overexpression, the immune-based interactions are dynamic and complex [25]. The move towards combining immune checkpoint inhibitors with chemotherapy and/or other novel agents may further limit the utility of PD-L1 expression. Therefore, additional studies are needed to establish reliable and dynamic predictive biomarkers that may vary across tumor type and indication. In the meantime, pathologists and oncologists must be careful to utilize the immune checkpoint inhibitors linked to PD-L1 status in the appropriate, FDA-approved setting.

## Supplementary information

**Additional file 1: Table S1.** Description of all U.S. FDA approvals of immune checkpoint inhibitors including date, drug information, indication, trial information (phase, endpoint leading to approval, PD-L1 cutoff) 

## Data Availability

All data generated or analyzed during this study are included in this published article and its supplementary information files.

## References

[1] Pardoll DM (2012). The blockade of immune checkpoints in cancer immunotherapy. Nat Rev Cancer.

[2] Patel SP, Kurzrock R (2015). PD-L1 expression as a predictive biomarker in Cancer immunotherapy. Mol Cancer Ther.

[3] Kythreotou A, Siddique A, Mauri FA, Bower M, Pinato DJ (2018). PD-L1. J Clin Pathol.

[4] Zhang J, Dang F, Ren J, Wei W (2018). Biochemical aspects of PD-L1 regulation in Cancer immunotherapy. Trends Biochem Sci.

[5] Sun C, Mezzadra R, Schumacher TN (2018). Regulation and function of the PD-L1 checkpoint. Immunity..

[6] Kowanetz M, Zou W, Gettinger SN, Koeppen H, Kockx M, Schmid P (2018). Differential regulation of PD-L1 expression by immune and tumor cells in NSCLC and the response to treatment with atezolizumab (anti-PD-L1). Proc Natl Acad Sci U S A.

[7] Mansfield AS, Aubry MC, Moser JC, Harrington SM, Dronca RS, Park SS (2016). Temporal and spatial discordance of programmed cell death-ligand 1 expression and lymphocyte tumor infiltration between paired primary lesions and brain metastases in lung cancer. Ann Oncol.

[8] Reck M, Rodriguez-Abreu D, Robinson AG, Hui R, Csoszi T, Fulop A (2016). Pembrolizumab versus chemotherapy for PD-L1-positive non-small-cell lung Cancer. N Engl J Med.

[9] Herbst RS, Baas P, Kim DW, Felip E, Perez-Gracia JL, Han JY (2016). Pembrolizumab versus docetaxel for previously treated, PD-L1-positive, advanced non-small-cell lung cancer (KEYNOTE-010): a randomised controlled trial. Lancet (London, England).

[10] Khozin S, Abernethy AP, Nussbaum NC, Zhi J, Curtis M, Tucker M (2017). Rates of PD-L1 expression testing in U.S. community-based oncology practices (USCPs) for patients with metastatic non-small cell lung cancer (mNSCLC) receiving nivolumab (N) or pembrolizumab (P). J Clin Oncol.

[11] Rosenberg JE, Hoffman-Censits J, Powles T, van der Heijden MS, Balar AV, Necchi A (2016). Atezolizumab in patients with locally advanced and metastatic urothelial carcinoma who have progressed following treatment with platinum-based chemotherapy: a single-arm, multicentre, phase 2 trial. Lancet (London, England).

[12] Bellmunt J, de Wit R, Vaughn DJ, Fradet Y, Lee JL, Fong L (2017). Pembrolizumab as second-line therapy for advanced Urothelial carcinoma. N Engl J Med.

[13] Stenehjem DD, Tran D, Nkrumah MA, Gupta S (2018). PD1/PDL1 inhibitors for the treatment of advanced urothelial bladder cancer. Onco Targets Ther.

[14] Chung HC, Ros W, Delord JP, Perets R, Italiano A, Shapira-Frommer R (2019). Efficacy and safety of Pembrolizumab in previously treated advanced cervical Cancer: results from the phase II KEYNOTE-158 study. Journal of clinical oncology : official journal of the American Society of Clinical Oncology.

[15] Fuchs CS, Doi T, Jang RW, Muro K, Satoh T, Machado M (2018). Safety and efficacy of Pembrolizumab Monotherapy in patients with previously treated advanced gastric and Gastroesophageal junction Cancer: phase 2 clinical KEYNOTE-059 trial. JAMA Oncol.

[16] Schmid P, Adams S, Rugo HS, Schneeweiss A, Barrios CH, Iwata H (2018). Atezolizumab and nab-paclitaxel in advanced triple-negative breast Cancer. N Engl J Med.

[17] Alexandrov LB, Nik-Zainal S, Wedge DC, Aparicio SA, Behjati S, Biankin AV (2013). Signatures of mutational processes in human cancer. Nature..

[18] Balar AV, Castellano D, O'Donnell PH, Grivas P, Vuky J, Powles T (2017). First-line pembrolizumab in cisplatin-ineligible patients with locally advanced and unresectable or metastatic urothelial cancer (KEYNOTE-052): a multicentre, single-arm, phase 2 study. Lancet Oncol.

[19] Carbone DP, Reck M, Paz-Ares L, Creelan B, Horn L, Steins M (2017). First-line Nivolumab in stage IV or recurrent non-small-cell lung Cancer. N Engl J Med.

[20] Sharma P, Retz M, Siefker-Radtke A, Baron A, Necchi A, Bedke J (2017). Nivolumab in metastatic urothelial carcinoma after platinum therapy (CheckMate 275): a multicentre, single-arm, phase 2 trial. Lancet Oncol.

[21] Hellmann MD, Nathanson T, Rizvi H, Creelan BC, Sanchez-Vega F, Ahuja A (2018). Genomic features of response to combination immunotherapy in patients with advanced non-small-cell lung Cancer. Cancer Cell.

[22] Samstein RM, Lee CH, Shoushtari AN, Hellmann MD, Shen R, Janjigian YY (2019). Tumor mutational load predicts survival after immunotherapy across multiple cancer types. Nat Genet.

[23] Gibney GT, Weiner LM, Atkins MB (2016). Predictive biomarkers for checkpoint inhibitor-based immunotherapy. Lancet Oncol.

[24] Cristescu R, Mogg R, Ayers M, Albright A, Murphy E, Yearley J (2018). Pan-tumor genomic biomarkers for PD-1 checkpoint blockade-based immunotherapy. Science (New York, NY).

[25] Hyman DM, Taylor BS, Baselga J (2017). Implementing genome-driven oncology. Cell..

