# Supplementary material for: The role of PD-L1 expression as a predictive biomarker: an analysis of all US Food and Drug Administration (FDA) approvals of immune checkpoint inhibitors
Source: J Immunother Cancer. 2019 Oct 26;7:278. doi: 10.1186/s40425-019-0768-9 (PMC6815032; doi:10.1186/s40425-019-0768-9)
Supplement: Supplementary file 1 — Additional file 1: Table S1. Description of all U.S. FDA approvals of immune checkpoint inhibitors including date, drug information, indication, trial information (phase, endpoint leading to approval, PD-L1 cutoff) [file 40425_2019_768_MOESM1_ESM.docx]

| **Year of approval** | **Immunotherapy Drug** | **Tumor Type** | **Histology (if applicable)** | **Indication** | **PMID/Name of Trial(s) Leading to Approval** | **Phase** | **Endpoint for approval** | **PD-L1 cutoff** | **FDA approval tied to PD-L1 assay** |
| --- | --- | --- | --- | --- | --- | --- | --- | --- | --- |
| 3/25/2011 | Ipilimumab | Skin | Melanoma | First line for BRAF V600 wild-type, unresectable or metastatic tumors | 20525992 | III | OS | not tested | no |
| 9/4/2014 | Pembrolizumab | Skin | Melanoma | Progression on ipilimumab and, if BRAF V600 mutation positive, a BRAF inhibitor | 25034862 | I | ORR | not tested | no |
| 12/22/2014 | Nivolumab | Skin | Melanoma | Progression on ipilimumab and, if BRAF V600 mutation positive, a BRAF inhibitor | 25795410 | III | ORR, response duration | 5% | no |
| 3/4/2015 | Nivolumab | Lung | NSCLC - squamous | Progression on platinum agents | 25704439 | II | OS | 5% | no |
| 9/30/2015 | Ipilimumab/Nivolumab | Skin | Melanoma | First line for BRAF V600 wild-type, unresectable or metastatic tumors | 25891304 | II | ORR, response duration, PFS | 5% | no |
| 10/2/2015 | Pembrolizumab | Lung | NSCLC | Progression on platinum agents for tumors expressing TPS > 50 | 25891174 | II | ORR | TPS > 50 | yes |
| 10/9/2015 | Nivolumab | Lung | NSCLC | Progression on platinum agents in tumors expressing TPS > 1 | 26412456 | III | OS | TPS > 1 | no |
| 10/28/2015 | Ipilimumab | Skin | Melanoma | Adjuvant treatment for fully resected stage III tumors | 25840693 | III | RFS | not tested | no |
| 11/23/2015 | Nivolumab | RCC | Clear cell | Progression on anti-angiogenic therapy | 26406148 | III | OS | 1%, 5% | no |
| 12/18/2015 | Pembrolizumab | Skin | melanoma | First line for unresectable or metastatic | 25891173, 26115796 | III | OS, PFS | 1% | no |
| 5/17/2016 | Nivolumab | lymphoma | Classical Hodgkin's | Progression after autologous HSCT and post-transplantation brentuximab vedotin | 25482239, 27451390 | II | ORR, response duration | no cutoff | no |
| 5/18/2016 | Atezolizumab | Bladder | Urothelial | Progression on platinum agents (locally advanced or metastatic) or within 12 months of neoadjuvant/adjuvant platinum agents | 26952546 | II | ORR, response duration | 5% | yes (b) |
| 8/5/2016 | Pembrolizumab | H&N | Squamous | Progression on platinum agents | 28328302 | II | ORR | TPS or CPS > 1 | no |
| 10/18/2016 | Atezolizumab | Lung | NSCLC | Progression on EGFR/ALK inhibitors (for patients with EGFR/ALK genomic aberrations) | 26970723, 27979383 | III | OS | 1%, 5%, 50% | no |
| 10/24/2016 | Pembrolizumab | Lung | NSCLC | First-line for patients with TPS > 50% and no EGFR/ALK aberrations | 26712084, 27718847 | III | OS, PFS | TPS > 50 | yes |
| 10/24/2016 | Pembrolizumab | Lung | NSCLC | Progression on platinum agents and/or EGFR/ALK inhibitors and PD-L1 > 1% | 26712084, 27718847 | III | OS, PFS | TPS > 1 | yes |
| 11/10/2016 | Nivolumab | H&N | Squamous | Progression on platinum agents | 27718784 | III | OS | 1%, 5%, 10% | no |
| 2/2/2017 | Nivolumab | Bladder | Urothelial | Progression on platinum agents (locally advanced or metastatic) or within 12 months of neoadjuvant/adjuvant platinum agents | 28131785 | II | ORR, response duration | 1%, 5% | no |
| 3/15/2017 | Pembrolizumab | Lymphoma | Hodgkin's lymphoma | Progression after > 3 lines of therapy | 28441111 | II | ORR, response duration | see comment (a) | no |
| 3/23/2017 | Avelumab | Skin | Merkel-cell carcinoma | First-line for patients ages 12+ | 27592805 | II | ORR, response duration | 1%, 5% | no |
| 5/1/2017 | Durvalumab | Bladder | Urothelial | Progression on platinum agents (locally advanced or metastatic) or within 12 months of neoadjuvant/adjuvant platinum agents | 28817753 | II | ORR, response duration | 25% | yes |
| 5/9/2017 | Avelumab | Bladder | Urothelial | Progression on platinum agents (locally advanced or metastatic) or within 12 months of neoadjuvant/adjuvant platinum agents | 29217288 | I | ORR, response duration | 5% | no |
| 5/10/2017 | Pembrolizumab | Lung | NSCLC - nonsquamous | First-line and in combination with carboplatin/pemetrexed | 27745820 | II | ORR, PFS, response duration | 1% | no |
| 5/18/2017 | Pembrolizumab | Bladder | Urothelial | Progression on platinum agents (locally advanced or metastatic) or within 12 months of neoadjuvant/adjuvant platinum agents | 28212060 | III | OS, ORR | CPS > 10 | yes (b) |
| 5/23/2017 | Pembrolizumab | Tissue agnostic |  | Mismatch repair deficient (dMMR) and/or microsatellite instability high (MSI-H) solid tumors with no treatment alternatives (for CRC after progression on 5-FU, oxaliplatin, irinotecan) | KEYNOTE-016, -164, -012, -028, -158 (MSI-H or dMMR cohorts) | II | ORR, response duration | not tested | no |
| 8/1/2017 | Nivolumab | Colon |  | dMMR and/or MSI-H with progression on 5-FU, oxaliplatin, irinotecan | 28734759 | II | ORR, response duration | 1% | no |
| 9/22/2017 | Nivolumab | Liver | HCC | Progression on sorafenib | 28434648 | II | ORR, response duration | 1% | no |
| 9/22/2017 | Pembrolizumab | Gastric/GE Junction | | Progression on 2+ systemic therapies (5-FU and platinum +/- Her2-targeted therapy) and CPS > 1 | 29543932 | II | ORR, response duration | 1% | yes |
| 12/20/2017 | Nivolumab | Skin | Melanoma | Lymph node involvement or resectable metastatic disease | 28891423 | III | RFS | 5% | no |
| 2/16/2018 | Durvalumab | Lung | NSCLC | Unresectable stage III whose disease has not progressed following concurrent platinum agents/radiotherapy | 28885881 | III | planned interim PFS | no cutoff | no |
| 4/16/2018 | Ipilimumab/Nivolumab | RCC | Clear cell | First line for Intermediate or poor risk | 29562145 | III | OS, ORR | 1% | no |
| 6/12/2018 | Pembrolizumab | Cervical |  | Progression on chemotherapy and CPS > 1 | 30943124 | II | ORR, response duration | 1% | yes |
| 6/13/2018 | Pembrolizumab | Lymphoma | Primary mediastinal B-cell | Progression after > 2 lines of therapy | KEYNOTE-170 | II | ORR, response duration | not listed | no |
| 7/10/2018 | Ipilimumab/Nivolumab | Colon |  | dMMR and/or MSI-H with progression on 5-FU, oxaliplatin, irinotecan | 29355075 | II | ORR, response duration | not listed | no |
| 8/16/2018 | Nivolumab | Lung | SCLC, extensive stage | Progression on platinum agents and at least one other line of therapy | 27269741 | II | ORR, response duration | 1%, 5% | no |
| 8/20/2018 | Pembrolizumab | Lung | NSCLC - nonsquamous | First line with no EGFR/ALK genomic aberrations and in combination with platinum agents/pemetrexed | 29658856 | III | PFS, OS | 1%, 50% | no |
| 9/28/2018 | Cemiplimab | Skin | SCC | First line for metastatic or locally advanced tumors not amenable to curative surgery or radiation | 29863979 | I | ORR, response duration | not tested | no |
| 10/30/2018 | Pembrolizumab | Lung | NSCLC - squamous | First line in combination with carboplatin and either paclitaxel or nab-paclitaxel | 30280635 | III | OS, PFS, ORR | 1% | no |
| 11/9/2018 | Pembrolizumab | Liver | HCC | Previously treated with sorafenib | 29875066 | II | ORR | 1% | no |
| 12/6/2018 | Atezolizumab | Lung | NSCLC - nonsquamous | Progression on platinum agents (and EGFR/ALK inhibitors if presence of molecular aberrations) | 29863955 | III | PFS, OS | no cutoff | no |
| 12/19/2018 | Pembrolizumab | Skin | Merkel-cell carcinoma | first line for recurrent locally advanced or metastatic | 27093365 | II | ORR, response duration | 1% | no |
| 2/15/2019 | Pembrolizumab | Skin | Melanoma | Adjuvant after resection of lymph node disease | 29658430 | III | RFS | 1% | no |
| 3/8/2019 | Atezolizumab | Breast | Triple negative | First line for unrsectable locally advanced or metastatic tumors expressing PD-L1 > 1% | 30345906 | III | PFS, ORR | 1% | yes |
| 3/18/2019 | Atezolizumab | Lung | SCLC, extensive stage | First line | 30280641 | III | PFS, OS | not tested | no |

Abbreviations: CPS = combined positive score, HCC = hepatocellular carcinoma, NSCLC = non-small cell lung cancer, ORR = overall response rate, PFS = progression-free survival, RFS = recurrence-free survival, SCLC = small-cell lung cancer, TPS = tumor proportion score

1. Three scores were reported separately: intensity score (0 to 3), membrane staining score (percentage of tumor cells with membrane staining; 0%, > 0 to < 50%, ≥ 50 to < 100%, or 100%), and histiocyte score (1 to 3; semiquantitative assessment of histiocytes/macrophages staining positive. Histiocyte staining was delineated from HRS cell staining by cytomorphologic assessment by the pathologist.
2. Companion PD-L1 testing approved only for cisplatin-ineligible patients with locally advanced/metastatic UC (Ventana SP142 (PD-L1 > 5%) treated with atezolizumab, Dako 22C3 assay CPS > 10 approved for patients on pembrolizumab). Platinum-ineligible patients regardless of PD-L1 status.
